# BRAF inhibitor resistance enhances vulnerability to arginine deprivation in melanoma

**DOI:** 10.18632/oncotarget.6882

**Published:** 2016-01-11

**Authors:** Ying-Ying Li, Chunjing Wu, Shu-Mei Chen, Sumedh S. Shah, Medhi Wangpaichitr, Lynn G. Feun, Macus T. Kuo, Miguel Suarez, Jeffrey Prince, Niramol Savaraj

**Affiliations:** ^1^ Sheila and David Fuente Graduate Program in Cancer Biology, University of Miami Miller School of Medicine, Miami, Florida, USA; ^2^ Sylvester Comprehensive Cancer Center, University of Miami Miller School of Medicine, Miami, Florida, USA; ^3^ Division of Hematology and Oncology, Miami Veterans Affairs Healthcare System, Miami, Florida, USA; ^4^ Graduate Institute of Clinical Medical Sciences, College of Medicine, Chang Gung University, Tao-Yuan, Taiwan; ^5^ Department of Neurosurgery, Taipei Medical University-Wan Fang Hospital, Taipei, Taiwan; ^6^ Dauer Electron Microscopy Laboratory, Department of Biology, University of Miami, Miami, FL, USA; ^7^ Department of Surgery, University of Miami Miller School of Medicine, Miami, Florida, USA; ^8^ Department of Molecular Pathology, University of Texas MD Anderson Cancer Center, Houston, Texas, USA; ^9^ Department of Laboratory Medicine, Miami Veterans Affairs Healthcare System, Miami, Florida, USA

**Keywords:** BRAF inhibitor resistance, arginine deprivation, autophagy, ASS1 re-expression, ubiquitin-proteasome machinery

## Abstract

BRAF inhibitor (BRAFi) has been used for treatment of melanomas harboring V600E mutation. Despite a high initial response rate, resistance to BRAFi is inevitable. Here, we demonstrate that BRAFi-resistant (BR) melanomas are susceptible to arginine deprivation due to inability to initiate re-expression of argininosuccinate synthetase (ASS1, a key enzyme for arginine synthesis) as well as ineffective autophagy. Autophagy and ASS1 re-expression are known to protect melanoma cells from cell death upon arginine deprivation. When melanoma cells become BR cells by long-term *in vitro* incubation with BRAFi, c-Myc-mediated ASS1 re-expression and the levels of autophagy-associated proteins (AMPK-α1 and Atg5) are attenuated. Furthermore, our study uncovers that downregulation of deubiquitinase USP28 which results in more active c-Myc degradation via ubiquitin-proteasome machinery is the primary mechanism for inability to re-express ASS1 upon arginine deprivation in BR cells. Overexpression of USP28 in BR cells enhances c-Myc expression and hence increases ASS1 transcription upon arginine deprivation, and consequently leads to cell survival. On the other hand, overexpression of Atg5 or AMPK-α1 in BR cells can redirect arginine deprivation-induced apoptosis toward autophagy. The xenograft models also confirm that BR tumors possess lower expression of ASS1 and are hypersensitive to arginine deprivation. These biochemical changes in BRAFi resistance which make them vulnerable to arginine deprivation can be exploited for the future treatment of BR melanoma patients.

## INTRODUCTION

BRAF mutation at V600E is present in 40-60% of cutaneous melanomas and can be treated with BRAF inhibitor (BRAFi) (vemurafenib or dabrafenib) [1, 2] or combined BRAFi and MEK inhibitor (MEKi). While the response rate is high (80%), resistance is inevitable and usually occurs within 12 months [3]. Hence, salvage therapy is urgently needed to treat these patients who failed BRAFi/MEKi. Immunotherapy, such as anti-CTLA4 antibody (ipillumamab) or anti-PD-1 antibody (nivolumab), has been shown to have anti-tumor activity in melanoma patients who failed chemotherapy, and triplet arm treatment (immunotherapy, BRAFi, and MEKi) has been done in a phase I clinical trial but was discontinued due to unacceptable gastrointestinal toxicity [4, 5]. The activity of these immunotherapies in BRAF/MEK inhibition failure remains unknown.

Currently, the major aberrant mechanisms leading to acquired resistance to BRAFi have been identified which include mutation of RAS, overexpression of CRAF, COT, PDGFRβ, and IGF-1R [6-9]. These mechanisms result in constitutive activation/phosphorylation of downstream molecules including ERK and AKT. Heterogeneous resistant mechanisms in the same tumor or different tumors in the same patient increase the difficulties to target a specific alternative pathway [10, 11]. In this study, we provide a different strategy to treat BR tumors by targeting the bioenergetic vulnerability shared by the majority of BR tumors regardless of the alternative pathways they use to circumvent BRAF inhibition.

Previously, we have reported that most melanoma cells (60-80%) do not express or express very low levels of argininosuccinate synthetase (ASS1) [12], a key enzyme in urea cycle needed to synthesize arginine from citrulline [13]. Hence, ASS1 negative (−) melanoma cells require exogenous arginine to maintain normal cellular function. We and others have shown that arginine deiminase (ADI-PEG20, kindly provided by Polaris Pharmaceuticals, Inc.), a mycoplasma enzyme degrading arginine to citrulline and ammonia, is active against multiple ASS1 (−) tumors including melanoma [13]. However, ASS1 re-expression and autophagy impair its antitumor activity [14, 15]. Combination treatments to abort autophagy and redirect the cells to apoptosis are being developed to circumvent these mechanisms [14, 16, 17].

Our previous study showed that ASS1 expression is positively regulated by c-Myc but negatively regulated by HIF-1α in melanoma cells [18]. Inducible ASS1 transcription upon arginine deprivation is primary a consequence of PI3K/AKT/ERK activation which enhances stability of c-Myc *via* downregulation of GSK-3β-phosphorylated c-Myc at Thr58 and upregulation of phosphorylated c-Myc (Ser62) [15, 19]. Additionally, a deubiquitinase, USP28, has been reported to antagonize ubiquitin-dependent proteasomal degradation of c-Myc. Elevated c-Myc overwhelms HIF-1α to bind E-box (enhancer box) in ASS1 promoter, and collaborates with transcription factor SP4 binding to GC box to initiate ASS1 transcription in melanoma cells [18]. When ASS1 is up-regulated, cells can synthesize arginine and not depend on exogenous arginine, resulting in ADI-PEG20 resistance.

Autophagy is known to emerge when cancer cells encounter nutrient stresses, chemotherapeutic agents, and protein kinase inhibitors [20] and is one of the major mechanisms leading to resistance. Arginine deprivation has been shown to induce autophagy through AMPK activation [21] which can negate its antitumor activity. Activated AMPK can directly activate ULK complex or through mTOR inhibition and in turn trigger formation of Atg-5-Atg12 complex and LC3-I/LC3-II conversion [12, 20, 22]. On the other hand, mutant BRAF (V600E) has been reported to constitutively phosphorylate ERK which can phosphorylate LKB1 directly or indirectly through ribosomal S6 kinase (RSK), and subsequently suppress LKB1 capability to activate AMPK in melanomas [23, 24]. AMPK protein *per se* can be degraded by ubiquitin-proteasome machinery [25]. Overall, the LKB1-AMPK axis, which is a master energy sensor regulating cell proliferation and survival through autophagy during nutrient stress, can be modulated by ERK activation and proteasomal degradation.

In this study, we found that BRAFi resistance abrogates ASS1 re-expression and autophagy, which are two vital mechanisms for survival when parental cells encounter arginine deprivation [18, 21]. Abrogation of ASS1 re-expression is most likely due to increased c-Myc degradation *via* ubiquitin-proteasome machinery, and downregulation of autophagy is due to a decrease in autophagy-associated proteins. Overall, these findings suggest that arginine deprivation/ADI-PEG20 can be applied as a salvage therapy for patients who fail BRAFi treatment.

## RESULTS

### BRAFi-resistant (BR) melanoma cells are more sensitive to arginine deprivation compared with parental cells

We have established BR cells from six parental cell lines (A375, A2058, MEL-1220, SK-MEL-28, MEL-GP, and UACC-62) which harbor BRAF (V600E) mutation. All parental cell lines were constantly exposed to vemurafenib at IC50 over 30 weeks. To confirm whether they become BRAFi resistant, both parental and BR cells were treated with different concentrations of vemurafenib for 72 hr, and IC50 values of BRAFi were assessed by MTT assay. The result revealed that IC50 values of BR cell lines were 2-10 fold higher than those of parental cell lines (Table 1).

**Table 1:** Synopsis of parental and BR melanoma cell lines

Cell line	AIC50 of vemurafnib ^a^	IC50 of ADI-PEG20 ^a^	Apoptotic cells ^b^ (%)
A375	0.5 ± 0.2 μM	181 ± 10.5 ng/ml	25.3 ± 2.5%
A375BR	10.2 ± 0.5 μM	68 ± 8.2 ng/ml	64.2 ± 5.9%
MEL-1220	4.2 ± 0.5 μM	205 ± 12 ng/ml	4.7 ± 2.2%
MEL-1220BR	15.2 ± 0.8 μM	62.5 ± 3.3 ng/ml	35.9 ± 4.5%
A2058	5.1 ± 0.4 μM	425 ± 18.5 ng/ml	5.1 ± 1.2%
A2058BR	25 ± 1.3 μM	125 ± 7.8 ng/ml	29.8 ± 3.3%
UACC-62	7.8 ± 0.6 μM	178 ± 7.3 ng/ml	7.7 ± 0.8%
UACC-62BR	17.2 ± 2.1 μM	92 ± 5.5 ng/ml	54.2 ± 3.2%
SK-MEL-28	2.5 ± 0.8 μM	222 ± 4.3 ng/ml	7.7 ± 3.5%
SK-MEL-28BR	10 ± 1.3 μM	103 ± 8.4 ng/ml	16.3 ± 2.8%
MEL-GP	0.8 ± 0.2 μM	> 1000 ng/ml	1.3 ± 0.5%
MEL-GPBR	4.3 ± 1.1 μM	> 1000 ng/ml	8.2 ± 1.2%

a IC50 values are based on cell viability curves performed by MTT assay.

b The percentages of apoptosis detected by Annexin V/PI were quantified by FACS following ADI-PEG20 treatment (100 ng/ml) for 72 hr. The proportions of treatment groups are normalized by untreated groups.

**Figure 1:**
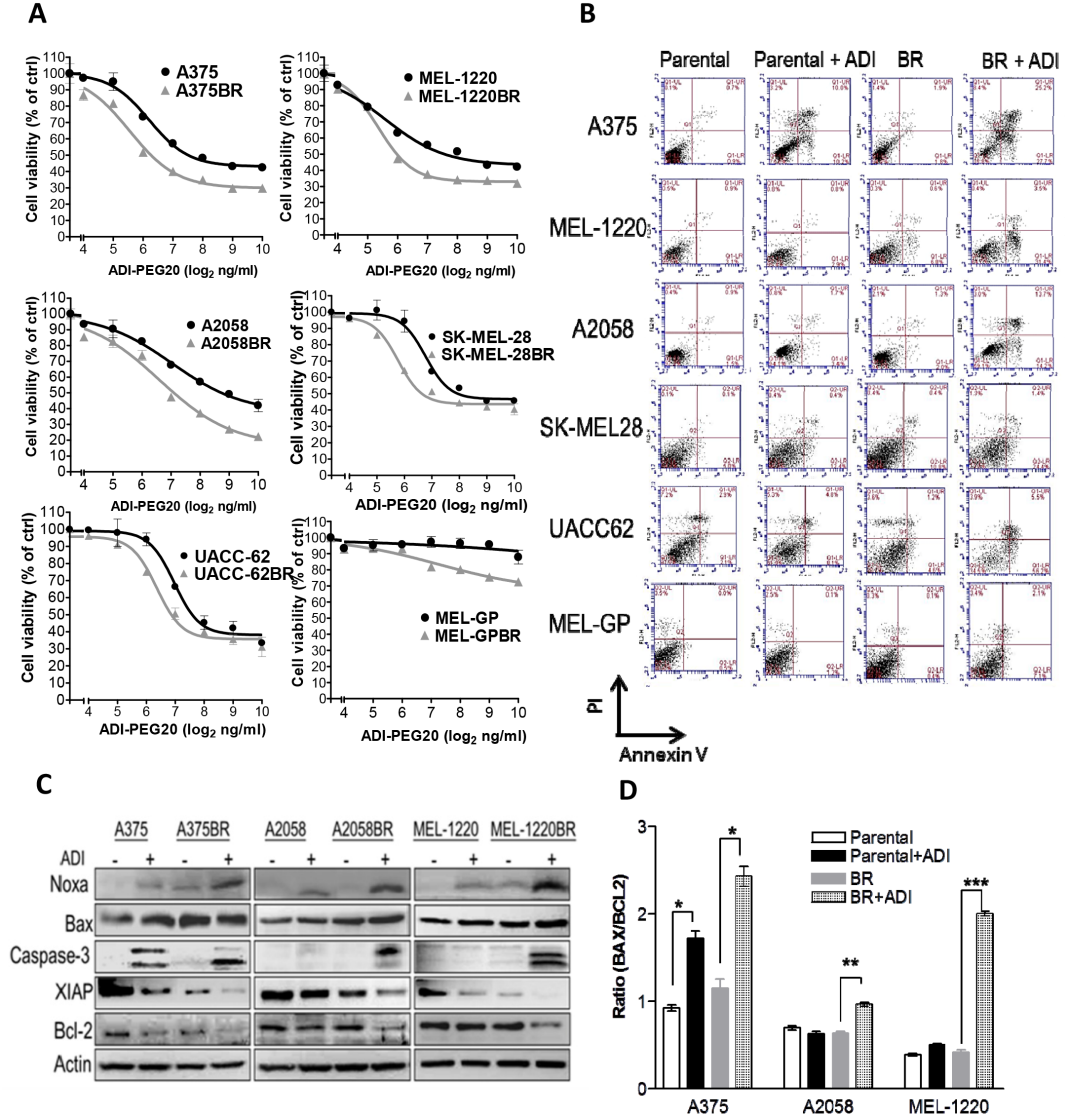
Arginine deprivation triggers caspase- dependent apoptosis in BR cells **A.** Cell viability was determined by MTT assay following treatment with ADI-PEG20 (0-1000 ng/ml) for 72 hr. **B.** Apoptotic proportions were analyzed by Annexin V/PI and FACS after treatment with ADI-PEG20 (100 ng/ml) for 72 hr. **C-D.** The levels of pro-apoptotic proteins and anti-apoptotic proteins were observed by immunoblotting and the BAX/BCL2 ratio was evaluated through densitometry. (*n* = 3, *p< 0.05, **p< 0.01, and ***p< 0.005).

To investigate whether BR cells are sensitive to arginine deprivation, parental and BR cells were treated with ADI-PEG20 (an arginine degrading enzyme) and cell viability was analyzed by MTT assay. As shown in Figure 1A and Table 1, the IC50 values of ADI-PEG20 of BR cell lines (A375BR, MEL-1220BR, A2058BR, SK-MEL-28BR, and UACC-62BR) were approximately 2.5-fold lower than their parental cell lines. The IC50 values of ADI-PEG20 in MEL-GP and MEL-GPBR were not reached due to the fact that MEL-GP has endogenous ASS1 as well as inducible ASS1 expression upon ADI-PEG20 treatment. Nevertheless, ADI-PEG20 was able to inhibit about 20% proliferation in MEL-GPBR cells but had no effect on parental cells (Figure 1A and Figure 5A). Overall, our data indicated that BR cells are hypersensitive to arginine deprivation/ADI-PEG20 treatment.

### Arginine deprivation induces apoptosis instead of autophagy in BRAFi-resistant (BR) cells

Since BR cells are hypersensitive to arginine deprivation, we proceeded to study whether cell death also occurs. Based on IC50 values of ADI-PEG20, we treated all cell lines with ADI-PEG20 (100 ng/ml) for 72 hr and then examined for apoptosis. The result depicted that ADI-PEG20 treatment notably showed an increase in Annexin V-positive BR cell proportion when compared to their parental cell counterparts (Figure 1B and Table 1). In addition, increased levels of cleaved caspase-3 were also detected in all three BR cells but only in one parental cell line A375, which is known to be sensitive to arginine deprivation (Figure 1C) [16, 21]. Addition of pan caspase inhibitor Z-VAD-FMK also rescued BR cells from ADI-PEG20-induced apoptosis (Supplementary Figure 1), and confirmed that ADI-PEG20 induced caspase-dependent apoptosis. We further examined the levels of pro-apoptotic (Noxa and Bax) and anti-apoptotic proteins (Bcl-2 and XIAP) which may contribute to the apoptotic effect of ADI-PEG20. These proteins were chosen since they have been shown to be affected by ADI-PEG20 treatment from our previous study [16]. The result revealed that the levels of Noxa were highest in all BR cells after ADI-PEG 20 treatment, whereas anti-apoptotic proteins declined in BR cells following ADI-PEG20 treatment (Figure 1C). Importantly, the Bax/Bcl2 ratio known to be regulated by Noxa and reflect BH3 protein ability to trigger caspase-3 activation and cytochrome *c* release [26] was significantly higher in BR cells compared to the untreated control and parental cells treated with ADI-PEG20 (Figure 1D). Thus, alterations of pro-apoptotic and anti-apoptotic proteins favoring apoptosis most likely contribute to the apoptotic effect of ADI-PEG20 in BR cells.

**Figure 2:**
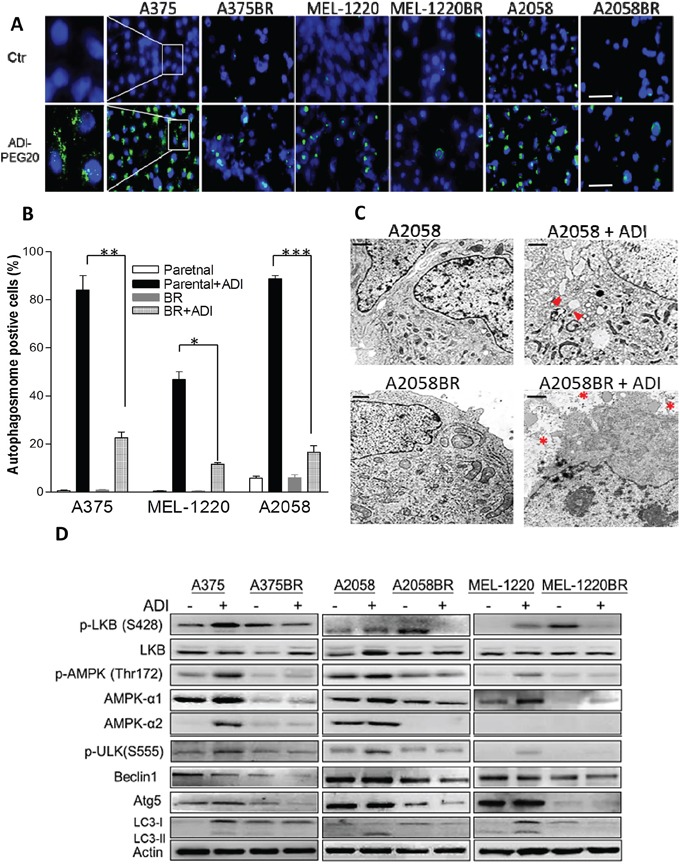
Low levels of autophagy-associated proteins are implicated in vulnerability to arginine deprivation in BR cells **A.** Autophagosomes and nuclei were respectively stained with Cyto-ID (green) and Hoechst33342 (blue) following incubation with arginine-free medium overnight, and then visualized by the fluorescent microscope (scale bar = 100 μm). **B.** The proportions of Cyto-ID positive cells are shown in a bar graph (*n* = 3, *p< 0.05, **p< 0.01, and ***p< 0.005. **C.** Autophagy (arrowheads) and apoptosis (asterisks) were visualized by the transmission electron microscope (TEM, scale bar = 0.5 μm). **D.** Cell lysates were extracted from parental and BR cells treated with or without ADI-PEG20 (100 ng/ml) for 72 hr, and the levels of autophagy-associated proteins were determined by immunoblotting.

Our previous studies demonstrated that ADI-PEG20 is able to trigger autophagy, which precludes parental melanoma cells from undergoing apoptosis and prolongs cell survival [14, 21]. To confirm whether ADI-PEG20 induces apoptosis by evading autophagy in BR cells, we compared the autophagosome formation and autophagy associated proteins in parental and BR cells upon arginine deprivation/ADI-PEG20 treatment. The result showed that ADI-PEG20 induced 45-90% autophagosome formation in parental cells but less than 25% autophagosome formation in BR cells (Figure 2A-B). The TEM images further depicted that ADI-PEG20 treatment resulted in increased numbers of autophagosomes in cytoplasm of A2058 cells (arrowheads), while organelle fragmentation and enlarged vacuoles which are indicative of apoptosis were seen in A2058BR cells (asterisks) (Figure 2C). Furthermore, another autophagic marker, conversion of LC3-I to LC3-II, was seen in parental cells after treatment with ADI-PEG20, but not in BR cells (Figure 2D, and Supplementary Figure 6). Taken together, our data confirmed that ADI-PEG20 induces autophagy in parental cells but apoptosis in BR cells.

### Downregulation of AMPK-α1 and Atg5 is crucial for vulnerability to arginine deprivation

**Figure 3:**
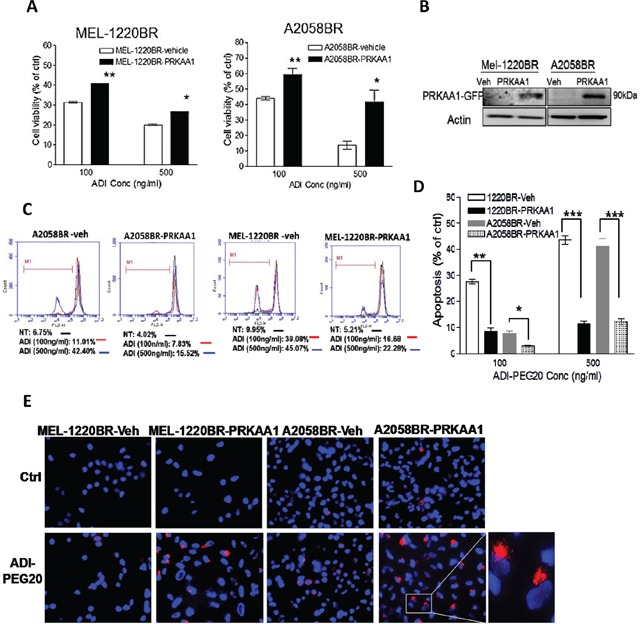
Overexpression of AMPKA-α1 (PRKAA1) switches ADI-PEG20-induced BR cell apoptosis toward autophagy **A.** MEL-1220BR and A2058BR cells were respectively transfected with plasmids containing PRKAA1-GFP, or with GFP (vehicle, veh). GFP-positive cells were incubated with completed medium (ctrl) or ADI-PEG20 (100 or 500 ng/ml) for 72 hr, and cell viability was detected by MTT. **B.** Overexpression of AMPK-α1 (PRKAA1) was confirmed by immunoblotting. **C-D.** The proportions of cell death were analyzed by TMRE and FACS and are shown in bar graphs after cultured in completed medium (ctrl) or ADI-PEG20 (100 or 500 ng/ml) for 72 hr. (n=3; *p< 0.05, **p< 0.01, and ***p< 0.005). **E.** Autophagosomes and nuclei were respectively stained with Lyso Tracker Red (red) and DAPI (blue), and then visualized by fluorescence microscopy.

We next investigated which autophagy-associated proteins dictate vulnerability to arginine deprivation. The data revealed that phosphorylation of AMPK (Thr172) and ULK (Ser555) were elevated in parental cells but attenuated in BR cells following ADI-PEG20 treatment (Figure 2D). It has been reported that BRAF-mediated ERK and RSK activation negatively regulates LKB through phosphorylation at Ser428, and subsequently inhibits activation and phosphorylation of AMPK [23]. However, our results showed that elevated p-LKB was seen in BR cells, yet attenuation of p-AMPK was still present and was not related to protein levels of p-LKB1 (Ser428) (Figure 2D). Instead, the levels of p-AMPK (Thr172) correlated with the levels of AMPK-α1 which possess (Thr172) phosphorylation site. Based on this result, we hypothesized that the inability to undergo autophagy following ADI-PEG20 treatment is primarily due to decreased levels of AMPK-α1. To verify this hypothesis, BR cells were transfected with the plasmids containing AMPK-α1 (PRKAA1) to compensate for insufficient endogenous AMPK-α1, and then treated with ADI-PEG20. The result revealed that overexpression of AMPK-α1 in MEL-1220BR and A2058BR cells resulted in robust autophagosome formation and decreased apoptosis as well as reduced growth inhibitory effect by 15-30% after ADI-PEG20 treatment compared to vehicle control (Figure 3A-E).

**Figure 4:**
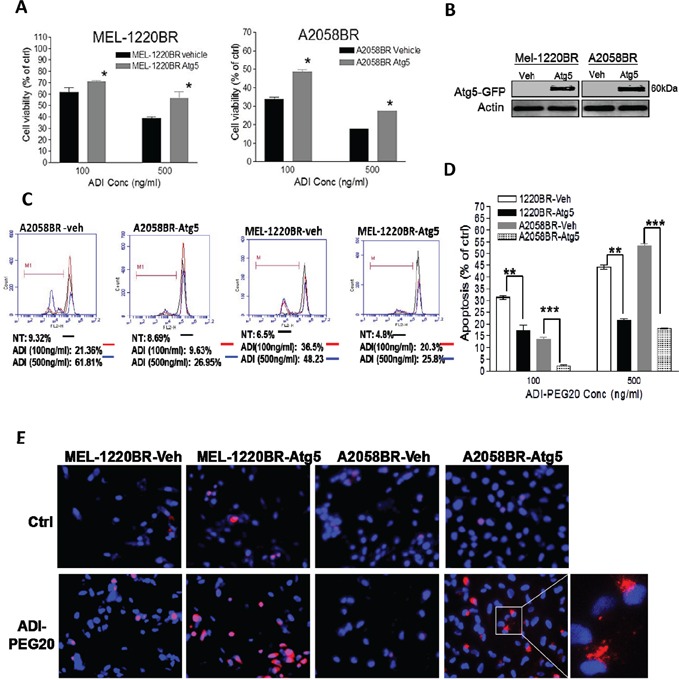
Overexpression of Atg5 also redirects ADI-PEG20-induced apoptosis toward autophagy in BR cells **A.** The plasmids containing Atg5-GFP and GFP (vehicle, veh) were delivered into MEL-1220BR and A2058BR, respectively. GFP-positive cells were sorted by FACS and incubated with completed medium (ctrl) or ADI-PEG20 (100 or 500 ng/ml) for 72 hr. Cell viability was detected by MTT. **B.** Overexpression of Atg5 was confirmed by immunoblot anlaysis. **C-D.** The apoptotic proportions were analyzed by TMRE and FACS and are shown in bar graphs following incubation with completed medium (ctrl) or ADI-PEG20 (100 or 500 ng/ml) for 72 hr. (n=3; *p< 0.05, **p< 0.01, and ***p< 0.005) **E.** Autophagosomes and nuclei were separately stained with Lyso Tracker Red (red) and DAPI (blue), and then visualized by fluorescence microscopy.

In addition to downregulation of AMPK-α1, the levels of downstream molecule of autophagy, Atg5, were also lower in BR cells compared to those in parental cells (Figure 2D). To examine whether Atg5 governs vulnerability to ADI-PEG20, MEL-1220BR and A2058BR cells were transfected with plasmids inserted with Atg5. As shown in Fig. 4A-D, over-expressed Atg5 increased 10-30% viability and rescued 12-35% apoptosis in BR cells through increased autophagy as evidenced by more autophagosome formation in BR cells following incubation with arginine-free medium (Figure 4E).

Previously, several studies have proven that BRAFi treatment can trigger ER stress-mediated autophagy which gives rise to BRAFi resistance [27], but BR cells created by long-term incubation with BRAFi in this study have insufficient AMPK-α1 and Atg5 and inactive autophagy. To verify this discrepancy, we did a 20-week time course study on the alterations of p-AMPK, AMPK-α1 and Atg5 after continuous exposure to BRAFi to mimic the clinical course when patients received BRAFi treatment. The results showed that p-AMPK, AMPK-α1, Atg5, and LC-3II increased during day 5 to day 15, yet they were attenuated after 55 days (Supplementary Figure 2). Notably, our time course study recapitulated the notion that these autophagy-associated proteins were down-regulated when melanoma cells entirely turned into BR cells.

### Downregulation of USP28 results in attenuation of c-Myc-mediated ASS1 transcription which increases sensitivity to arginine deprivation in BR cells

**Figure 5:**
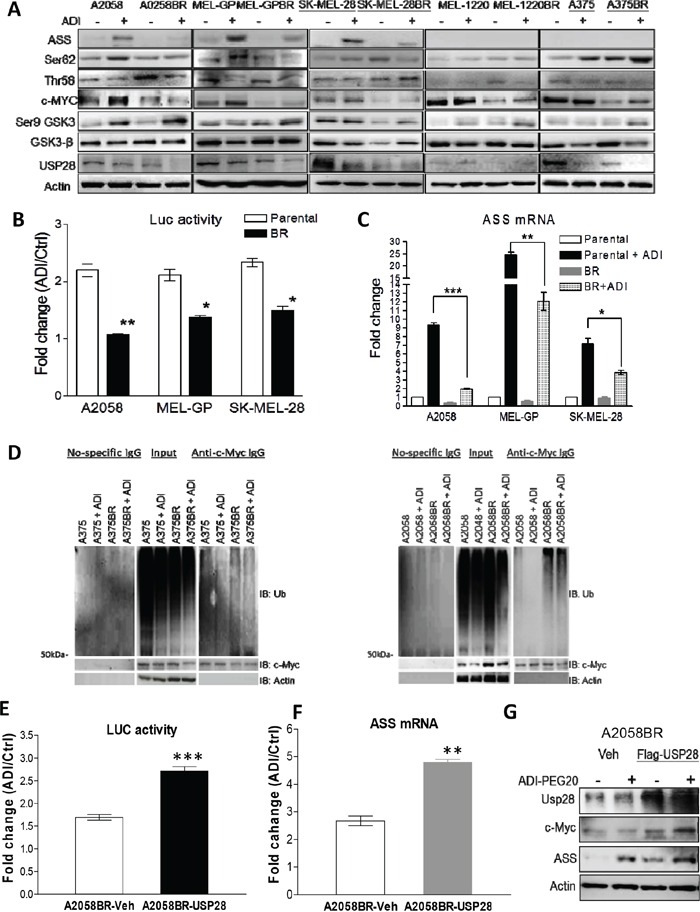
Down-regulated USP28 abrogates c-Myc-mediated ASS1 re-expression in BR cells **A.** Cell lysates from A2058, MEL-GP, and SK-MEL28 (inducible ASS1), and A375 and MEL-1220 (non-inducible ASS1) treated with ADI-PEG20 (100 ng/ml) for 48 hr were subjected to immunoblotting analysis. **B.** The activities of c-Myc binding to ASS1 promoter were represented as luciferase activity normalized by protein concentration. The PGL3 plasmids containing ASS1 promoter and luciferase gene were delivered into the cells, and activity of luciferase was analyzed after incubation with completed medium (ctrl) or arginine-free medium for 24 hr. **C.** The mRNA levels of ASS1 were determined by qRT-PCR after treatment with ADI-PEG20 (100 ng/ml) for 48 hr. (*n* = 3, *p< 0.05, **p< 0.01, and ***p< 0.005). **D.** Parental and BR cells were incubated with or without ADI-PEG20 in the presence of MG-132 (10 μM) for 4 hr. Ubiquitin (Ub) and c-Myc were respectively detected by immunoblotting following immunoprecipitation (IP) of c-Myc. **E-F.** Overexpression of Flag-USP28 in A2058BR cells enhanced c-Myc-mediated ASS1 transcription after incubation with ADI-PEG20 (100 ng/ml) for 48-72 hr. **G.** Immunoblotting confirmed overexpressed Flag-USP28 increased c-Myc and ASS1 levels.

As mentioned, c-Myc mediated ASS1 re-expression is a major contributory factor hampering the antitumor effect of ADI-PEG20. As shown in Figure 5A and 5C, following ADI-PEG20 treatment, ASS1 and c-Myc levels were notably robust in parental A2058, MEL-GP, and SK-MEL-28 cells, but decreased in BR cells. To substantiate this finding, we determined activity of c-Myc binding to promoter (E-box) of ASS1 with the plasmid PGL3 inserted with E-box and luciferase gene. As shown in Figure 5B, a 2-2.5-fold increase in induced luciferase activity was seen in parental cells, whereas only a 1.2-1.5-fold increase was seen in BR cells. In parental cell lines (A375 and MEL-1220) with non-inducible ASS1 expression, c-Myc protein levels were increased upon ADI-PEG20 treatment; however, attenuated c-Myc levels were still seen when they became BR cells (Figure 5A and Supplementary Figure 6). Furthermore, the results of time course experiment also reiterated that both ASS1 and c-Myc were down-regulated simultaneously in A2058 cells after exposure to BRAFi (Supplementary Figure 2). Previous data showed that ASS1 induction upon ADI-PEG20 treatment is mediated through PI3K/Akt and ERK activation in A2058 cells [15]. Activated Akt phosphorylates GSK-3β at Ser9 and inhibits its ability to phosphorylate c-Myc at Thr58 (the site inducing c-Myc degradation). Meanwhile, activated ERK directly phosphorylates c-Myc at Ser62 to stabilize c-Myc [15, 18]. Consistent with previous study, ADI-PEG20 treatment increased the ratio of Ser62 to Thr58 and in turn enhanced c-Myc stability leading to ASS1 expression in parental cells. In BR cells, low levels of c-Myc did not correlate with p-c-Myc (the ratio of Ser62/Thr58), and GSK-3β (Figure 5A). Thus, other factors may be involved in governing c-Myc stability. Our previous study showed that HIF-1α competes with c-Myc to bind to ASS1 promoter and impedes its transcription [18]. To clarify whether HIF-1α suppresses ASS1 transcription in BR cells, we treated both parental and BR cells with hypoxia-mimetic cobalt chloride (CoCl_2_) or proteasome inhibitor MG-132 to abort HIF-1α degradation. The results revealed that HIF-1α expression was too low to terminate ASS1 transcription (Supplementary Figure 3). Taken together, our data demonstrated that suppressed ASS1 transcription is mainly mediated by attenuated c-Myc in BR cells.

Since low c-Myc levels seen in BR cells may be due to decreased transcription, we detected RNA levels using qRT-PCR and found no association with protein levels (data not shown). Thus, we hypothesized that more active ubiquitin-proteasome machinery of these proteins occurs in BR cells. To validate this hypothesis, we determined the levels of these proteins upon treatment with proteasome inhibitor MG-132 or a protein synthesis inhibitor cycloheximide (CHX). The results showed that all three BR cell lines had delayed accumulation of c-Myc following treatment with MG-132 (Supplementary Figure 4). Subsequent to treatment with CHX, c-Myc levels declined faster in BR cells than parental cells. To further determine whether more active ubiquitination of these two proteins appears in BR cells, ubiquitin co-immunoprecipitated with c-Myc was detected by immunoblotting. The results showed that higher levels of ubiquitin binding to c-Myc are present in BR cells, relative to parental cells, even in the presence of ADI-PEG20 (Figure 5D).

It has been reported that USP28 is a potent deubiquitinase to facilitate c-Myc stabilization [28], and down-regulated USP28 appeared in all BR cells (Figure 5A and Supplementary Figure 6). Hence, we hypothesized that attenuation of USP28 may play a role in c-Myc degradation seen in BR cells. To examine this hypothesis, the plasmid containing Flag-USP28 was delivered into A2058BR cells. Overexpression of USP28 resulted in an increase in c-Myc protein levels due to less ubiquitination and proteasomal degradation (Supplementary Figure 5). Increased c-Myc bound to promoter of ASS1 gene (ASS1 transcription) and resulted in increased ASS1 protein in A2058BR-USP28 cells following treatment with ADI-PEG20 (Figure 5E-G).

### *In-vivo* models confirm arginine deprivation-induced apoptosis and attenuated ASS1 re-expression in BR tumors

**Figure 6:**
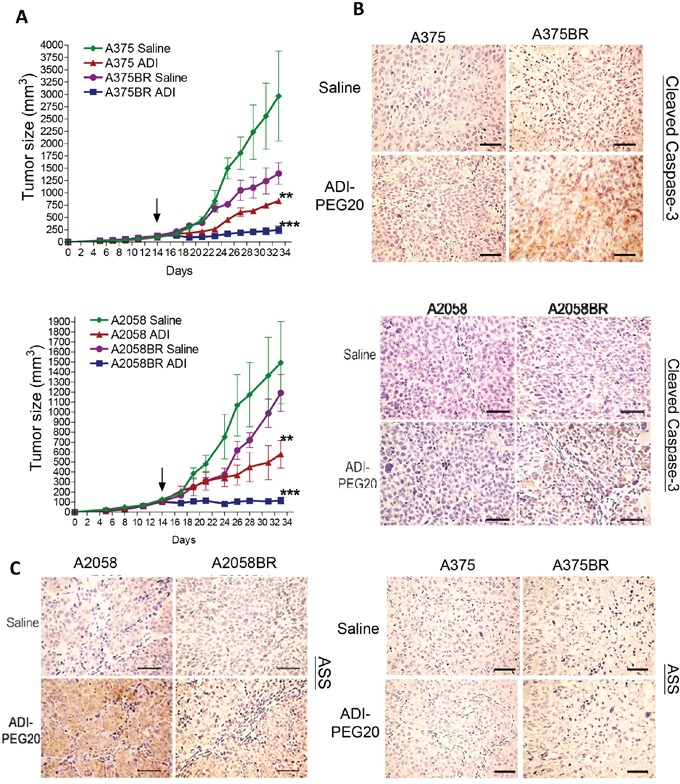
ADI-PEG20 aborts BR tumor growth through increased cleavage of caspase-3 and attenuation of ASS1 expression **A.** 1 × 10^6^ melanoma cells were subcutaneously inoculated into nude mice. When tumor volume reached 100 mm^3^ (on day 14, black arrow), tumor bearing mice were treated with ADI-PEG20 (100 IU/kg) twice per week. Tumor volume was represented as mean + SEM (*n* = 5; *p< 0.05, **p< 0.01, and ***p< 0.005). Xenograft tumors were obtained form A375 and A375BR tumor bearing mice. **B-C.** The levels of ASS1 and cleaved caspase-3 were detected in A2058/A2058BR and A375/A375BR xenograft tumors using IHC staining and visualized by light microscopy (scale bar = 100 μm).

The results of xenograft model showed that ADI-PEG20 treatment retarded tumor growth of A2058 and A375 cells, and entirely aborted tumor growth in A2058BR and A375BR cells (Figure 6A). The T/C (treatment/control) ratios in A2058BR and A375BR cells were lower than those in A2058 and A375 cells (A375 vs. A375BR is 26.7% vs. 11.8%; A2058 vs. A2058BR is 38.7% vs. 8.3%). Furthermore, elevated cleaved caspase-3 expression was also found in tumor tissues from A375BR- and A2058BR-bearing mice treated with ADI-PEG20 (Figure 6B). Importantly, consistent with our *in-vitro* data, the attenuated ASS1 re-expression was present in A2058BR xenograft tumors and ASS1 expression was negative in A375/A375BR xenograft tumors (Figure 5A and 6C).

### BRAFi/MEK inhibitor (MEKi) dual resistance sensitizes melanoma cells to arginine deprivation as well as BRAFi resistance

The combination of BRAFi and MEKi has been used to treat melanoma patients and results in dual resistance. As shown above, BR cells are hypersensitive to ADI-PEG20 treatment due to their impaired ability to re-express ASS1 and undergo autophagy. Whether BRAFi/MEKi resistant (BMR) cells also share similar biochemical changes which make them vulnerable to ADI-PEG20 treatment is unknown. To investigate this possibility, we have established BMR cells from parental cells (A2058BMR), and study ASS1 expression and sensitivity to ADI-PEG20 treatment. Similar to BR cells, attenuated ASS1 and AMPK-α1 expressions were also seen in BRAFi/MEKi resistant (BMR) cells (Figure 2D and 5A, and Supplementary Figure 7). Importantly, ADI-PEG20 treatment induced 35-45% apoptosis instead of autophagy, without induction of ASS1 (Supplementary Figure 7). Moreover, the levels of USP28 and c-Myc were also attenuated in BMR cells. Thus, our results demonstrated that arginine deprivation therapy can also be applied to treat BMR melanomas.

## DISCUSSION

At present, there is no FDA approved drug to treat patients who failed BRAFi or BRAFi/MEKi. Targeting alternative pathways in BR tumors is also difficult due to tumor heterogeneity and the response may also be short-lived [10]. Non-specific inhibitors such as HSP90 inhibitor and CDK4/6 inhibitor have been shown to have antitumor activity in BR tumors *in vitro* and in animal models [29-31], but the clinical activity is as yet unknown. Recently it was reported that ER stress-mediated autophagy is also a mechanism of BRAFi resistance [32]. Combination of BRAFi with hydroxychloroquine (autophagy inhibitor) is suggested to further improve the therapeutic efficacy of BRAFi [27]. Similar to these findings, our time course study shows that melanoma cells undergo autophagy as a survival mechanism when they first encounter BRAFi (Supplemental Figure 2). Nevertheless, we found that BRAFi initially triggers autophagic flux and then attenuates the levels of autophagy-associated proteins (AMPK-α1, Atg5, and LC3-II) over time. This result suggests that these cells initiate proliferation and hence abandon autophagy after they completely adapt to inhibitors. Importantly, it also corresponds to the clinical finding that patients who failed BRAFi treatment often have rapid disease progression [7, 11].

In this report, we have discovered that all BR cells are exquisitely sensitive to arginine deprivation and arginine deprivation treatment leads to apoptosis instead of autophagy through multiple mechanisms. Firstly, attenuation of autophagy occurs in BR cells as mentioned above. Secondly, BR cells have elevated levels of the pro-apoptotic protein Noxa and low levels of anti-apoptotic proteins including XIAP and Bcl-2 when treated with ADI-PEG20 (Figure 1C), which favors apoptosis. Thirdly, ASS1 re-expression upon ADI-PEG20 treatment is down-regulated which results in vulnerability to ADI-PEG20 treatment. Thus, we have utilized several experiments to elucidate the underlying mechanisms.

ASS1 transcription is governed by many mechanisms and may depend on tumor cell types. Epigenetic ASS1 gene silencing via DNA methylation has been reported in mesothelioma and other cancers [33, 34] and is not inducible. The mechanisms leading to methylation of ASS1 gene remain unknown. In melanoma, ASS1 transcription is primarily governed by c-Myc (a positive regulator) and HIF-1α (a negative regulator) and is inducible in parental cells in arginine depleted conditions [18]. However, in certain melanoma cell lines, such as MEL-1220 cells, epigenetic silencing does play a role in ASS1 expression as evidenced by the result that non-inducible ASS1 is seen in parental cells. On the other hand, in A375 cells, the tight binding of HIF-1α, which cannot be displaced by c-Myc, does play an important role in non-inducible ASS1[18]. In contrast to parental cells, BR cells have very low levels of both HIF-1α and c-Myc. The lower levels of HIF-1α also correspond with published data which indicate that BRAFi treatment drives the cells toward oxidative phosphorylation [35]. Unlike parental cells, the protein levels of c-Myc in BR cells are so low that they cannot efficiently upregulate ASS1 transcription even under arginine deprivation. Attenuation of c-Myc is primarily due to a decrease in deubiquitination and is not related to two phosphorylation sites (Thr58/Ser62). Two deubiquitinases (USP28 and USP36) have been reported to govern c-Myc stability [28, 36]. We found a substantial reduction in USP28 with no significant difference in USP36 between parental and BR cells (data not shown). Currently, the mechanism that USP28 interacts with c-Myc and antagonizes c-Myc degradation is controversial. Several studies suggested that certain types of ubiquitin ligase (Fbw7) are recruited by phosphorylation of c-Myc at Thr58, bridge USP28 and c-Myc interaction, and enhance the ability of USP28 to remove the ubiquitin from c-Myc [28, 37]. However, our finding shows that down-regulation of USP28 in BR cells attenuates c-Myc stability in a phosphorylation (Thr58/Ser62) site-independent manner, which is in agreement with other studies supporting that USP28 can directly bind to c-Myc in the absence of F-box protein Fbw7 [38]. Additionally, according to the study by Popov, et al., depletion of USP28 in HeLa and LS174T cells can inhibit cell growth and proliferation due to its inability to enhance c-Myc stability [28]. This finding may explain why BR tumors *in vivo* grow more slowly than their parental tumors (Figure 6A). Taken together, USP28 is likely to govern not only ASS1 re-expression but also proliferation in BR cells. Downregulation of c-Myc via proteasomal regulation in BR cells may also have other metabolic consequence and correlate with the recent data showing that BR cells use oxidative phosphorylation rather than glycolysis as their energy source [39].

The energy sensing LKB1-AMPK axis has been shown to be suppressed by BRAF-ERK activation [23, 24]. In BRAF (V600E) mutant melanoma, two phosphorylation sites of LKB (Ser325 and Ser 428), which are crucial for regulation of AMPK activation, are constitutively phosphorylated by ERK and RSK. However, upon treatment with BRAFi, p-AMPK is activated and autophagy occurs most likely through inhibition of ERK and as a mechanism to evade apoptosis (Supplementary Figure 2). As discussed earlier, it has been shown that BRAFi triggers autophagy via ER stress [27]. It is possible that inhibition of ERK and activation of ER stress contribute to autophagy upon treatment with BRAFi. When melanoma cells become BRAFi resistant, lower levels of p-AMPK may be governed by downregulation of AMPK-α1. Our data also demonstrate that overexpression of AMPK-α1 in BR cells indeed can reduce apoptosis and increase autophagy. This finding supports the notion that down-regulated AMPK-α1 mainly influences phosphorylation of AMPK (Thr172) (Figure 2D and Figure 3) which regulates autophagy. Additionally, we also assayed RNA levels of AMPK-α1 in both parental and BR cells and found no correlation with protein levels (data not shown). These results suggest that downregulation of AMPK- α1 in BR cells may be through ubiquitin-dependent proteasomal degradation.

In addition to down-regulated AMPK seen in BR cells, Atg5 is also attenuated in BR cells, which can impair elongation of autophagic membrane. On the other hand, Atg5 can be cleaved by calpain and trigger apoptosis [40, 41]. We found only full length of Atg5 (32KDa) but not cleaved Atg5 (∼24KDa) is down-regulated in BR cells, even in the presence of ADI-PEG20. Overexpressed Atg5 can partially restore ADI-PEG20-induced autophagy and prevent BR cells from apoptosis (Figure 4). This is expected since AMPK levels remain low in these Atg5 transfected cells.

**Figure 7:**
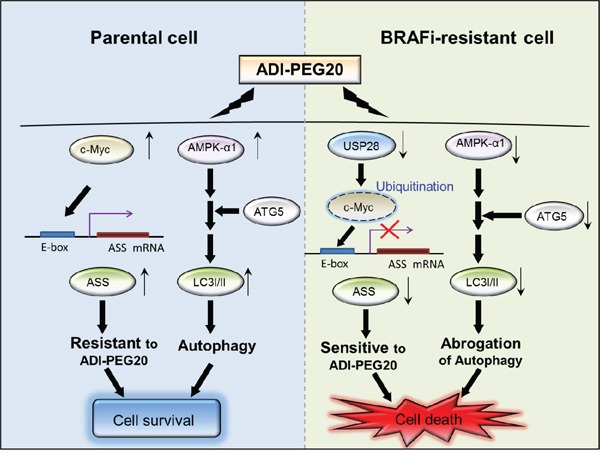
The schematic diagram illustrates the two mechanisms leading to sensitivity to arginine deprivation/ADI-PEG20 In parental cells, ASS1 re-expression and autophagy attenuate ADI-PEG20 efficacy resulting in cell survival. In BR cells, downregulation of c-Myc via ubiquitin-proteasome degradation and attenuated AMPK-α1 respectively result in inability to initiate ASS1 transcription and undergo autophagy, and hence increase sensitivity to ADI-PEG20. Downregulation of USP28 is accountable for c-Myc degradation and suppresion of ASS1 transcription.

In summary, melanoma cells, in the process of protecting themselves from the cytotoxic effect of BRAFi, first utilize autophagy to survive and gradually initiate alternative pathways to activate ERK and/or other downstream growth signaling pathway(s). Once they survive and become BR cells, they start to proliferate and abrogate autophagic flux by decreasing AMPK-α1 and Atg5. These alterations together with the inability to turn on ASS1 expression make them extremely vulnerable to arginine deprivation (Figure 7). It is possible that the cells in tumor microenvironment, such as endothelial cells, can provide arginine and abrogate the effect of ADI-PEG20; however, hypersensitivity to ADI-PEG20 is clearly seen *in vivo* which suggests that tumor microenvironment may not play a major role in abrogating ADI-PEG20 effect seen in BR cells. Overall, our data elucidate that degrading arginine using ADI-PEG20 could be a promising non-toxic salvage therapy in both BRAFi and/or MEKi- resistant melanomas.

## MATERIALS AND METHODS

### Cell lines and reagents

The BRAFi-resistant (BR) cell lines were established from SK-MEL28, MEL-GP, A375, MEL-1220, UACC-62 and A2058 cells possessing V600E mutation. The parental cell lines including A375, A2058, UACC62, and SK-MEL28 were obtained from American Type Culture Collection (ATCC), and MEL-1220 and MEL-GP were established in our laboratory. To establish BR cells, these parental cells were incubated with BRAFi (vemurafenib, PLX4032) or BRAFi/MEKi (trametinib, GSK1120212) (Selleck Chemicals) at IC50 over 30 weeks. All cell lines were cultured in MEM supplemented with 10% FBS (Atlanta Biologicals, Inc) and streptomycin/penicillin in CO_2_ incubator. The arginine-free medium was generated by adding ADI-PEG20 (100 ng/ml, Polaris Pharmaceuticals, Inc.) in complete MEM medium at 37°C over 24 hr.

To investigate the mechanism of proteasomal degradation, we treated the parental and BR cells with either a proteasome inhibitor MG-132 (Selleck Chemicals) or an inhibitor of protein synthesis (cyclohexmide, Sigma) to observe the turnover of c-Myc. Hypoxia mimetic agent CoCl2 (Sigma-Aldrich) was prepared in complete medium and used to treat the cells for detection of HIF-1α expression.

### Antibodies for immunoblotting

Antibodies against HIF-1α, c-Myc, USP28, ubiquitin (Ub), AMPK-α1, AMPK-α2, AMPK, phospho-AMPK (Thr172), Caspase-3, GSK-3β, phospho-GSK-3β (Ser9), XIAP, Beclin-1, p-LKB (S428), ULK (S555) and LC3-I/II were purchased from Cell Signaling Technology. The rest of the antibodies, such as anti-Atg5, anti-phospho-c-Myc (Thr58), anti-phospho-c-Myc (Ser62), LKB and anti-ASS1 were respectively purchased from Abgent, Santa Cruz Biotech, Abcam, and kindly provided by Polaris Pharmaceuticals, Inc. The immunoblots were visualized by ChemiDoc MP System (Bio-Rad) and quantitation was performed by densitometer and Image J.

### BRAF mutation analysis

Total DNA was isolated from melanoma cells using DNeasy blood & tissue kit (Qiagen). The BRAF mutation in DNA samples (50 ng/μl) was determined using BRAF V600E mutation analysis kit (EntroGen) and iCycler iQ™ real-time PCR detection system (CFX96, Bio-Rad). The protocol for detection followed the instructions of the manufacturer.

### Reverse transcription and real-time PCR (qRT-PCR) analysis

For RNA detection, total RNA was extracted by trizole reagent (Life Technologies) and converted into cDNA using iScript cDNA synthesis kit (Bio-Rad). The cDNA was subjected to real-time PCR analysis for detection of AMPK-α1, and c-Myc using SYBR green supermix reagent and real-time PCR machine (CFX96, Bio-Rad). The levels of these genes were normalized by GAPDH levels. Their primer sequences were described in supplementary Table 1.

### Transfection of plasmids and promoter/luciferase activity assays

Plasmid PGL-AS-85 was provided by Dr. Macus T. Kuo [18]. Luciferase activity assay was accomplished by cells transfected with mixture of plasmid PGL3-AS-85 (containing E-box) and lipofectamine (Invitrogen) for 6 hr and then cultured in arginine-free medium for 24 hr. The activity of c-Myc binding to promoter was determined by luciferase assay system kit (Promega) and normalized by total protein. Overexpression of USP28 was accomplished using a plasmid containing Flag-USP28 (pDZ-Flag-USP28) (Addgene, Inc). The GFP containing plasmids inserted with Atg5, or AMPK-α1 (PRKAA1) were purchased from Addgene and OriGene, respectively. Subsequent to transfection of these plasmids with lipofectamine, GFP-positive cells were sorted by FACS Arial I sorter (BD Biosciences).

### Cell viability assay

Prior to ADI-PEG20 treatment, BR melanoma cells were cultured in the absence of vemurafenib for 24 hr. 5 × 10^3^ melanoma cells were cultured with various doses of ADI-PEG20 for 72 hr. The cell proliferation was analyzed by MTT (Sigma-Aldrich), which has been described in previous study [42].

### Analyses of apoptosis and autophagy

Apoptosis was detected using Annexin V and PI kit (Abd Serotec), and tetramethylrhodamine, ethyl ester dye (TMRE, Sigma-Aldrich) according to the manufacturer's instruction. The apoptotic proportion was analyzed by Accuri™ C6 flow cytometer (BD Biosciences). To confirm whether ADI-PGE20-induced cell apoptosis is caspase dependent, the cells were treated with pan-caspase inhibitor Z-VAD-FMK (20 μM, Santa Cruz) in the presence of ADI-PEG20, stained with Annexin V/PI, and analyzed by flow cytometry FACS. For detection of autophagosomes, cyto-ID autophagy detection kit (Enzo Life Sciences) and Lyso Tracker Red (Life Technologies) were applied to this experiment.

### Immunoprecipitation

Briefly, immunoprecipitation was completed by addition of anti-c-Myc (Cell Singling Technology) and Gammabind plus sepharose bead slurry (GE Healthcare) into the protein samples. Immunoprecipitants were collected and analyzed by SDS-PAGE and immunoblotting.

### Xenograft study

The procedures and protocol of mice were approved by the Institutional Animal Care and Use Committee (IACUC) of Miami VA medical center. Female athymic nude-Foxn1^nu^ mice (6-8 weeks, Harlan Laboratories) were inoculated subcutaneously with 1 × 10^6^ cells prepared in physiologic buffered saline (PBS), respectively. When the tumor volumes reached 100 mm^3^, the treatment group received intramuscular injection of ADI-PEG20 (100 IU/kg) twice per week, and control group was saline only. The formula of tumor volume was (length x width^2^)/2. Tumor growth inhibition was calculated as median of tumor volume in treatment group to that in control group (T/C) ratio. NCI standard of the T/C ratio is < 42% which indicates significant tumor growth inhibition.

### Immunohistochemical (IHC) staining

Tumor samples were immersed in 10% formalin for 2 week followed by 70% ethanol for a few days, and then dehydrated with gradient concentrations of ethanol and embedded in paraffin blocks. The tissue slides (4 μm) were dewaxed by xylene. Antigen retrieval was carried out with citric acid (10 mM, pH 6.0) containing 0.05% Tween 20.

For IHC staining, the tumor tissue slides were separately hybridized with anti-cleaved caspase-3 antibodies (Cell Singling Technology, 1:200) and anti-ASS1 antibodies (Polaris, 1:50) at 4°C overnight. Thereafter, the slides were stained with LSAB™2 Kits (DAKO) and hematoxylin (DAKO) and visualized by a light microscope (Olympus).

### Transmission electron microscopy (TEM)

Cell pellets were acquired and immediately fixed in neutral buffered 2.5% glutaraldehyde at room temperature. The specimens were post-fixed in 1% osmium tetroxide (OsO_4_) for 10 minutes, dehydrated using a graded ethanol series, en bloc stained with 2% uranyl acetate in 50% ethanol for 30 minutes, and embedded in Spurr's epoxy resin. Tumor samples were also embedded in a similar fashion described for cell cultures, except post-fixation staining with OsO_4_ and embedding with Spurr's resin was lengthened to account for the larger cell volume in tumor tissue. Semi-thin (1 μm) and ultra-thin (< 90 nm) sections were cut using a Diatome 3 mm diamond knife on the Leica EM UC6 ultramicrotome. Semi-thin sections were stained with toluidine blue and examined using an Olympus BX60 light microscope with a digital camera (Olympus DP71). All ultra-thin sections were stained using lead citrate to be viewed under TEM with a Jeol 1400 EM at 80 kV.

### Statistics

Statistical analysis was done by Student's *t*-test using Excel 2010 (Microsoft) and IC50 was assessed using Prism. All results of *in-vitro* studies were shown as mean ± standard error of mean (SEM). Every result was performed by three independent experiments. *P*-value < 0.05 was regarded as significant difference.

## SUPPLEMENTARY FIGURES AND TABLE


