# Supplementary material for: BRAF inhibitor resistance enhances vulnerability to arginine deprivation in melanoma
Source: Oncotarget. 2016 Jan 11;7(14):17665–80. doi: 10.18632/oncotarget.6882 (PMC4951241; doi:10.18632/oncotarget.6882)
Supplement: Supplementary file 1 [file oncotarget-07-17665-s001.pdf]

BRAF inhibitor resistance enhances vulnerability to arginine deprivation in melanoma

SUPPLEMENTARY FIGURES AND TABLE

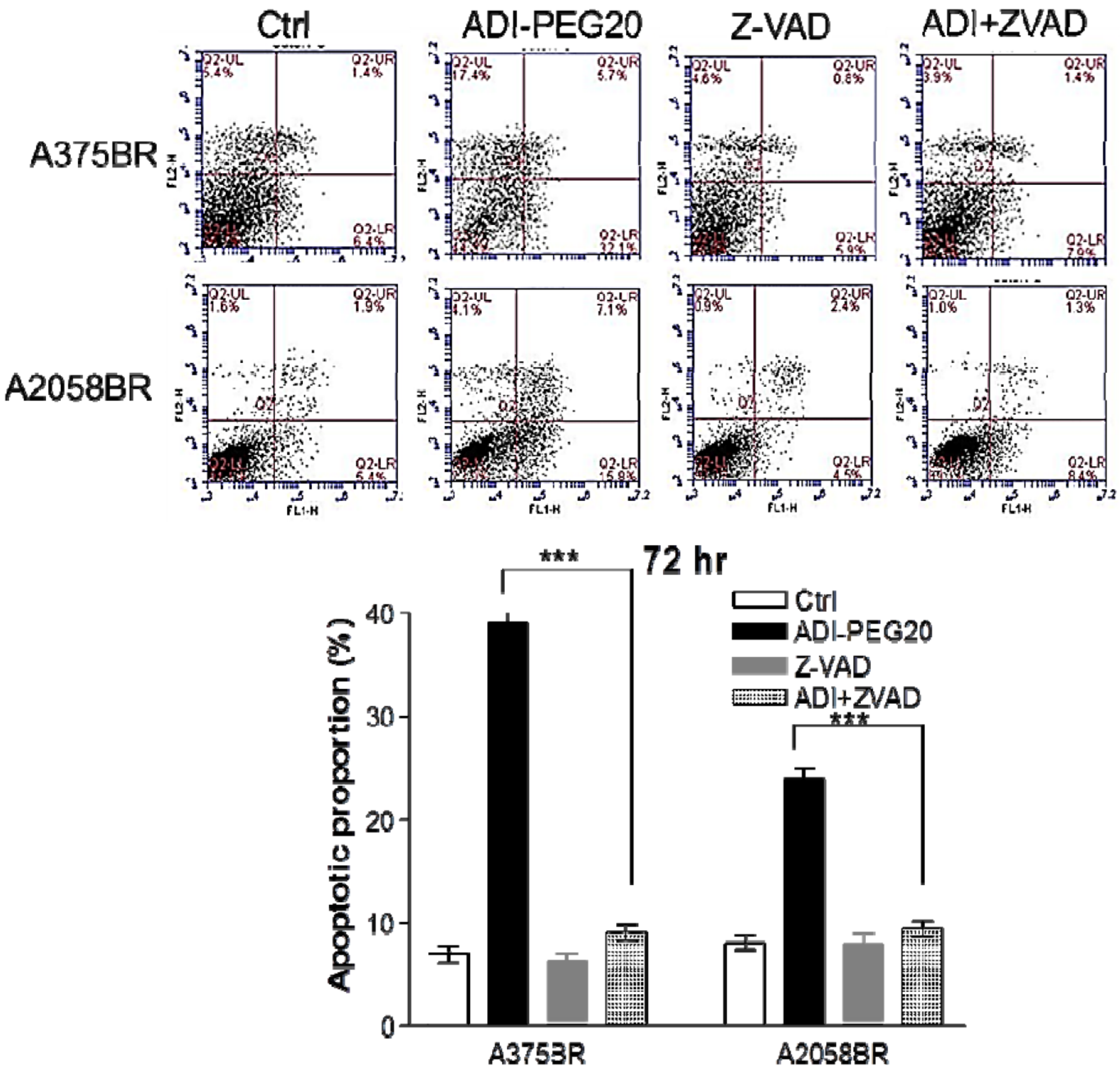

Supplementary Figure S1: Pan caspase inhibitor can rehabilitate BR cell viability following treatment with ADI-PEG20. A375BR and A2058BR cells were respectively incubated with completed medium (ctrl), ADI-PEG20 (100 ng/ml), pan caspase inhibitor Z-VAD-FMK (20 µM), and combination for 72 hr. Apoptotic cells were stained with Annexin V/PI and analyzed by FACS. A bar graph exhibits apoptotic proportions (Annexin V positive cells) (**p*< 0.05, ***p*< 0.01, and ****p*< 0.005).

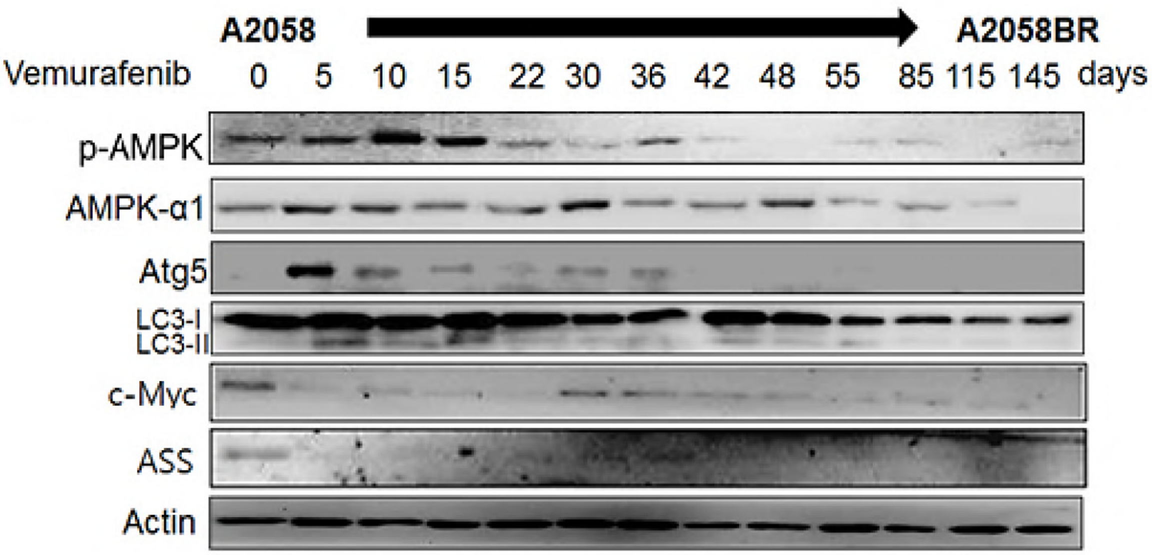

Supplementary Figure S2: The levels of autophagy- or ASS expression- associated proteins alter over time after exposure to BRAFi. A2058 cells were treated with vemurafenib (5 µM) for 145 days. Cell lysates were collected at different time points and protein levels were analyzed by immunoblotting.

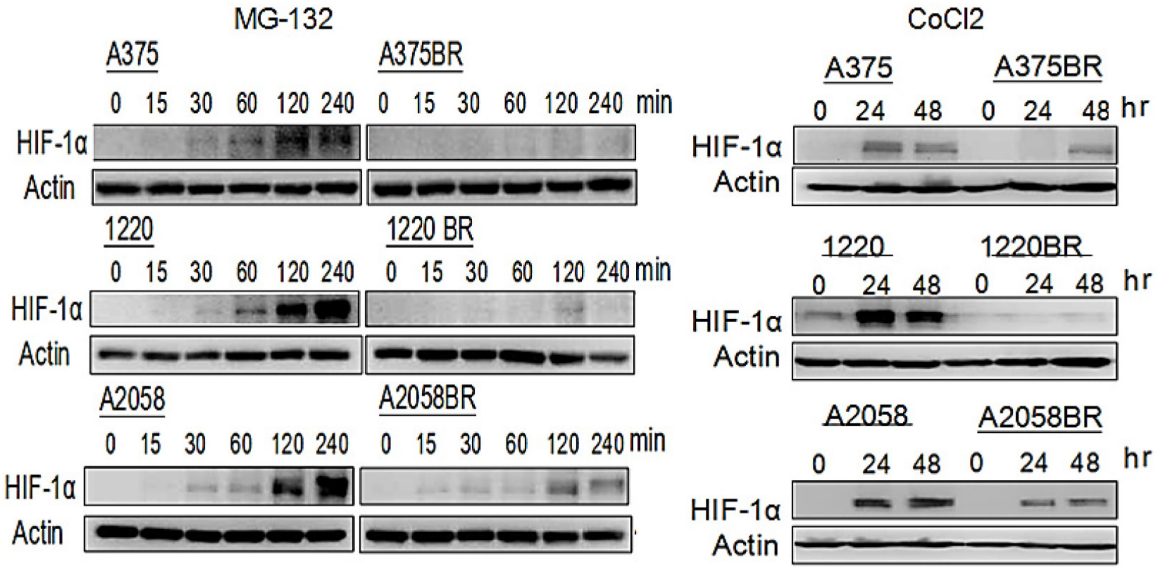

Supplementary Figure S3: Lower levels of HIF-1α appear in BR cells relative to parental cells. Three pairs of melanoma cell lines (parental and BR cells) were treated with proteasome inhibitor MG-132 (10 µM) or $CoCl_2$ (100 µM) to prevent HIF-1α degradation, and cell lysates were collected at different time points and then subjected to immunoblotting analysis.

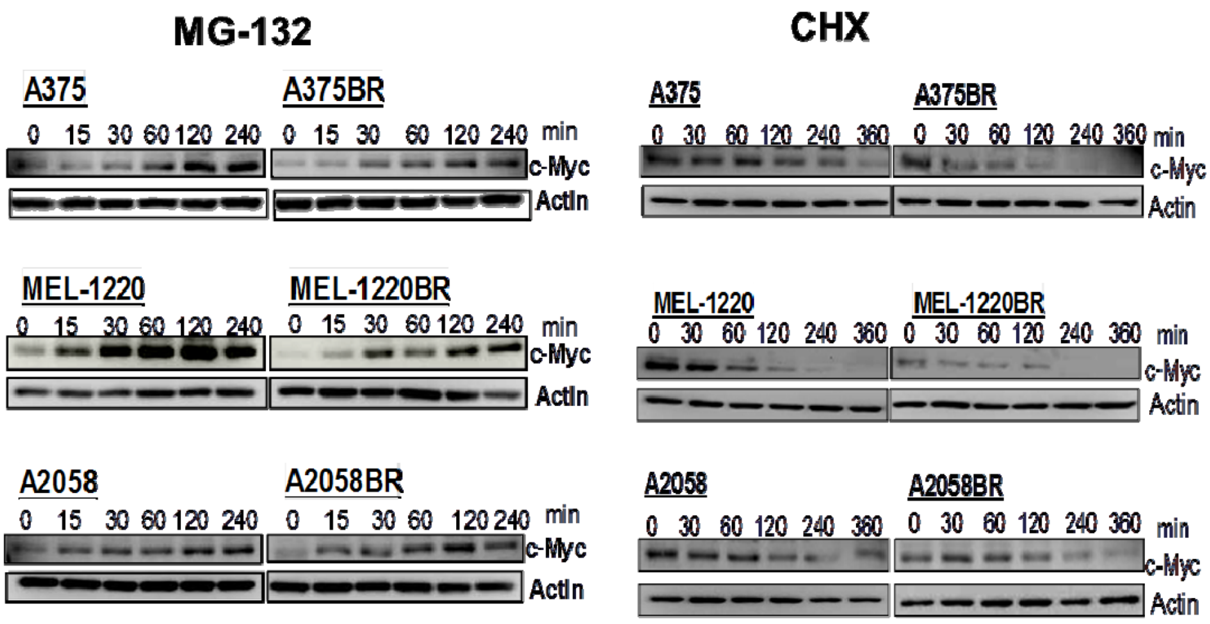

Supplementary Figure S4: More active proteasomal degradation of c-Myc is present in BR cells. Parental cell and BR cells were incubated with MG-132 (10 µM) or CHX (80 µg/ml). Parental and BR cell lysates were collected at different time points, and assayed by immunoblotting simultaneously.

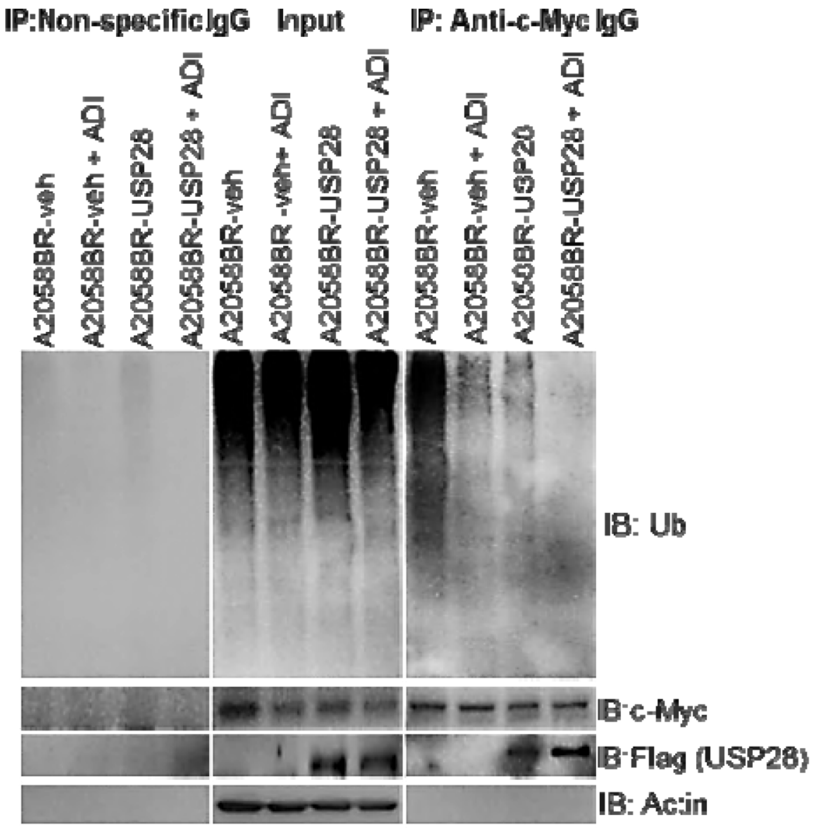

Supplementary Figure S5: Overexpression of USP28 attenuates ubiquitination of c-Myc. After transfected with plasmids containing USP28-Flag, A2058BR cells were treated with or without ADI-PEG20 in the presence of MG-132 (10 μM) for 4 hr. USP28-Flag and ubiquitin (Ub) in A2058BR cells lysates were co-immunoprecipitated with c-Myc and then detected by immunoblotting. A2058BR-veh represented a negative control.

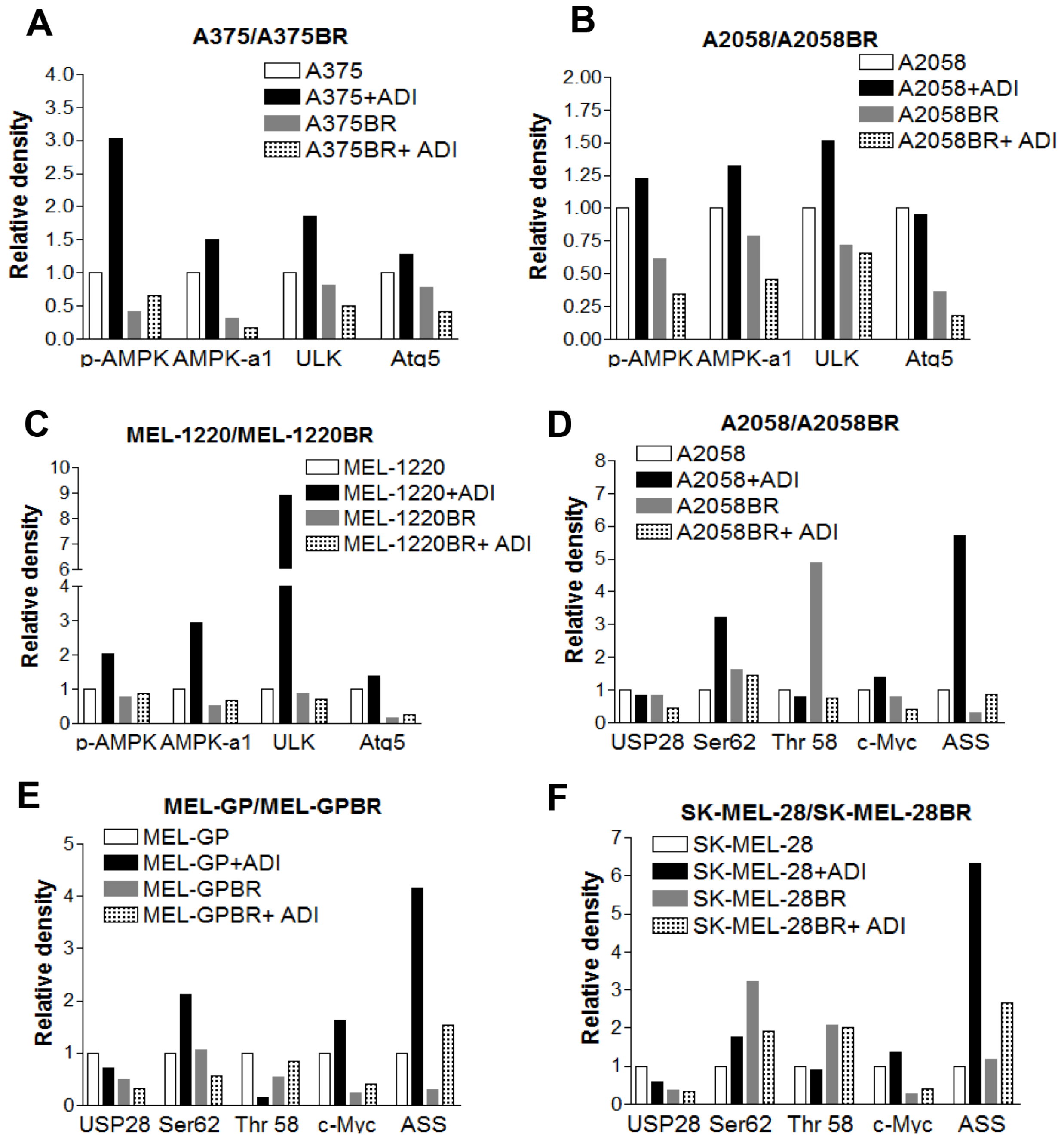

Supplementary Figure S6: The intensity of immunoblot has been quantitated and represented by bar graphs. A-C. The bar graphs represented the intensity of p-AMPK, AMPK-α1, ULK(S555), and Atg5 on immunoblot shown in Figure 2D. **D-F.** The bar graphs represented the intensity of USP28, c-Myc (Ser62), c-Myc (Thr58), c-Myc, and ASS shown in Figure 5A. A2058, MEL-GP, and SK-MEL-28 cell lines had inducible ASS expression upon ADI-PEG20 treatment. The intensity of these proteins was normalized by actin and parental cells without ADI-PEG20 treatment.

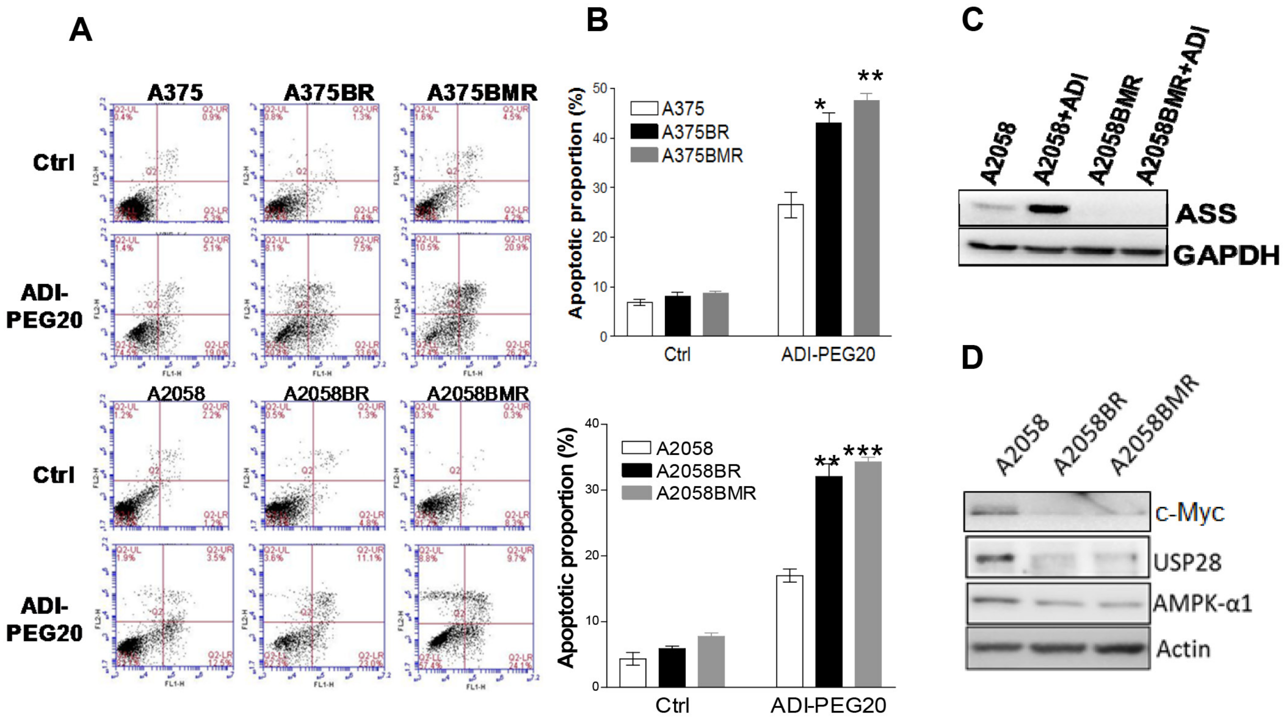

Supplementary Figure S7: BRAFi/MEKi resistant (BMR) cells show vulnerability to ADI-PEG20 as well as BR cells. A. BR and BMR cells respectively represented BRAFi resistant and BRAFi/MEKi resistant cells which survived in the presence of BRAFi or BRAFi/MEKi at IC50 over 30 weeks. These cells were treated with 100 ng/ml ADI-PEG20 for 72 hr, labeled with Annexin V/PI, and then analyzed by FACS. **B.** The bar graphs represent the percentages of Annexin V positive cells (*$p< 0.05$, **$p< 0.01$, and ***$p< 0.005$). **C.** Protein levels of ASS were determined in A2058 and A2058BMR cells treated with or without ADI-PEG20 (100 ng/ml) for 72 hr. **D.** Downregulation of AMPK-α1, c-Myc, and USP28 appeared in BR and BMR cells.

Supplementary Table 1: Primer sequences for qRT-PCR

Gene	Forward primer (5'→3)	Reverse primer (5'→3')
AMPK-α1 (PRKAA1)	GGTCCATAGAGATTTGAAACCTG	GCCTGCATACAATCTTCCTG
c-Myc	TCAAGAGGCGAACACACAAC	GGCCTTTTCATTGTTTTCCA
ASS	AACTCACGCCTCCAATCC	CATAGCCTTGTTCCTTCAGC
GAPDH	CTCTCTGCTCCTCCTGTTC	GGTGTCTGAGCGATGTGG
